# Identification of nuclear membrane SUN proteins and components associated with wheat fungal stress responses

**DOI:** 10.1007/s44154-024-00163-z

**Published:** 2024-06-11

**Authors:** Huan Guo, Jianfeng Wang, Di Yao, Ligang Yu, Wenting Jiang, Lincai Xie, Shikai Lv, Xiangyu Zhang, Yajuan Wang, Changyou Wang, Wanquan Ji, Hong Zhang

**Affiliations:** 1 State Key Laboratory for Crop Stress Resistance and High-Efficiency Production, College of Agronomy, Northwest A & F University, Yangling, Shaanxi 712100 China; 2 State Key Laboratory for Crop Stress Resistance and High-Efficiency Production, College of Plant Protection, Northwest A & F University, Yangling, Shaanxi 712100 China; 3 Engineering Research Center of Wheat Breeding, Ministry of Education, Yangling, Shaanxi 712100 China

**Keywords:** *Triticum aestivum*, SUN, KASH, Fungal stress

## Abstract

In eukaryotes, the nuclear membrane that encapsulates genomic DNA is composed of an inner nuclear membrane (INM), an outer nuclear membrane (ONM), and a perinuclear space. SUN proteins located in the INM and KASH proteins in the ONM form the SUN-KASH NM-bridge, which functions as the junction of the nucleocytoplasmic complex junction. Proteins containing the SUN domain showed the highest correlation with differentially accumulated proteins (DAPs) in the wheat response to fungal stress. To understand the characteristics of SUN and its associated proteins in wheat responding to pathogen stress, here we investigated and comprehensive analyzed SUN- and KASH-related proteins among the DAPs under fungi infection based on their conserved motifs. In total, four SUN proteins, one WPP domain-interacting protein (WIP), four WPP domain-interacting tail-anchored proteins (WIT), two WPP proteins and one Ran GTPase activating protein (RanGAP) were identified. Following transient expression of *Nicotiana benthamiana*, TaSUN2, TaRanGAP2, TaWIT1 and TaWIP1 were identified as nuclear membrane proteins, while TaWPP1 and TaWPP2 were expressed in both the nucleus and cell membrane. RT-qPCR analysis demonstrated that the transcription of TaSUN2, TaRanGAP2 and TaWPP1 were strongly upregulated in response to fungal infection. Furthermore, using the bimolecular fluorescence complementation, the luciferase complementation and a nuclear and split-ubiquitin-based membrane yeast two-hybrid systems, we substantiated the interaction between TaSUN2 and TaWIP1, as well as TaWIP1/WIT1 and TaWPP1/WPP2. Silencing of TaSUN2, TaRanGAP2 and TaWPP1 in wheat leaves promoted powdery mildew infection and hyphal growth, and reduced the expression of TaBRI1, TaBAK1 and Ta14-3–3, indicating that these NM proteins play a positive role in resistance to fungal stress. Our study reveals the characteristics of NM proteins and propose the preliminary construction of SUN-WIP-WPP-RanGAP complex in wheat, which represents a foundation for detail elucidating their functions in wheat in future.

**Supplementary Information:**

The online version contains supplementary material available at 10.1007/s44154-024-00163-z.

## Introduction

In eukaryotes, genomic DNA is enclosed by the nuclear envelope (NE), which consists of an inner nuclear membrane (INM) and an outer nuclear membrane (ONM), with the perinuclear space (PNS) enclosed by these two membranes. Nuclear pore complexes (NPC) embedded in the NE form channels connecting the nucleoplasm and cytoplasm. At the basic structural level, the NE functions as a barrier that protects the genome. However, the multifunctional nature of the NE was recognized recently (Zhou et al. 2015a). For instance, the NE harbors transmembrane proteins (NETs) that play important roles in nuclear structure, mechano-transduction, genome organization, gene regulation, cell polarization, and cell migration (Mekhail and Moazed 2010; Zhou et al. 2012b, 2015b). The constitutive expresser of pathogenesis-related genes 5 (CPR5), an inhibitor of programmed cell death (PCD) and effector-triggered immunity (ETI), was described as a nucleoporin (Nup) (Gu et al. 2016). Unlike Nups, the INM Sad1/UNC-84 (SUN) proteins cooperate with Klarsicht/ANC-1/Syne-1 homology (KASH) proteins as ONM-binding partners to form SUN-KASH NE bridges (Zhou et al. 2012b) that are also known as “linkers of the nucleoskeleton and the cytoskeleton (LINC) complexes” (Crisp et al. 2006). LINC complexes not only play important roles in nuclear localization, nuclear shape maintenance, pollen nucleus movement, stomatal movement, and male fertility of plants (Graumann et al. 2014; Tatout et al. 2014; Evans et al. 2020; Groves et al. 2020), but are also involved in seed maturation and germination, organ development, stress response and regulation of gene activity. Recently, *Arabidopsis thaliana* WPP domain-interacting proteins (AtWIPs) and SUN-interacting NE proteins (SINEs) were identified as plant-specific KASH proteins (Zhou et al. 2012a, 2014). The WPP domain is a protein domain comprising approximately 90 amino acid (aa) residues with a highly conserved tryptophan-proline-proline motif. The WPP domain-containing proteins, including Ran GTPase activating protein (RanGAP), MFP1 (a filament-like protein binding matrix attachment region DNA) attachment factor 1 (MAF1) and WPPs, are associated with the NE by protein–protein interactions (Meier 2000; Patel et al. 2004) and function in the nucleocytoplasmic shuttle (Zhang et al. 2022). WIPs also interact with WPP domain–interacting tail-anchored proteins (WITs), and function synergistically to anchor RanGAP1 to the NE (Zhao et al. 2008).

Wheat (*Triticum aestivum* L.) is grown on more land than any other crop worldwide, and wheat-based foods provide a significant source for about 40% of the world’s population human kind (Savary et al. 2019). However, wheat production was seriously threatening by biotrophic fungal diseases, such as powdery mildew (caused by *Blumeria graminis* f. sp. *Tritici, Bgt*) and stripe rust (caused by *Puccinia striiformis* f. sp. *tritici, Pst*). In practice, the cultivation of disease-resistant wheat varieties emerges as the safest, most cost-effective method to control disease. Consequently, exploring resistance (R) genes and understanding mechanisms had become a crucial task in present. Improvements in genome resources and functional research tools over the last two decades have facilitated great progress in the identification of host resistance genes, pathogen virulence genes and their interactions (Saur and Huckelhoven 2021).

During the highly dynamic plant-pathogen interaction process, the localization of more and more R proteins and pathogen effectors indicated that the nuclear transport of proteins plays an important role in plant defense responses. For example, the AVR_A10_ binds to the *Bgt-*specific immunity-related MLA10 receptor in barley to promote its entry into the nucleus, where it is then able to trigger an immune response (Halterman et al. 2001; Shen et al. 2007). AtNPR1 contains a bipartite nuclear localization signal that is inactive in the cytoplasm in an oligomeric state, while NPR1 is transformed into a monomeric state that enters the nucleus to activate resistance factors in response to pathogen infection. The nuclear accumulation of NPR1 in the nucleus requires the nuclear membrane protein NUP88/MOS7 (Mou et al. 2003; Cheng et al. 2009). In addition, MOS7 promotes the nuclear retention of MPK3 and plays a role in defense signal transduction against the necrotrophic fungus *Botrytis cinerea* (Genenncher et al. 2016). The Lamin-like LINC1 protein, which interacts with SUN proteins, plays a key role in JA signaling and regulation of PTI responses in Arabidopsis (Jarad et al. 2019). Taken together, these reports indicated that the nuclear membrane facilitates spatial isolation of transcription factors or transcriptional regulators and the signal-dependent nuclear input, representing important aspects of the activation of defense-related genes. However, full details of the function or activation of NETs and related components in wheat, particularly in defending to biotic and abiotic stress, remain to be established.

Since the basic principles of plant–microbe interactions are undoubtedly applicable to interactions between *Triticeae* and fungal pathogens, we focused on the role of KASH proteins and their interacting proteins in the mechanism by which SUN domain-containing protein 2-like (TraesCS1B02G108700) participates in the response of wheat to fungal stress (Fu et al. 2016). Based on detailed knowledge of the KASH domains and characterization of the WPP and conserved methionine-leucine-glutamic-X(n)-leucine-glutamic-lysine (MLEXnLEK) motifs of WITs, we identified several putative KASH-interacting proteins in wheat. Furthermore, we analyzed the primary functional characterization of five candidates from three protein subfamilies and demonstrated their interactive relationship with seven NM proteins, as well as their functions in wheat against powdery mildew (*Blumeria graminis* f. sp. *Tritici, Bgt*) infection, to help clarify the role of the KASH subset of plant NETs in plant defense against fungal stress. This study reveals the structural characteristics of some classic NM proteins, and constructs a possible composite model by their interactions, which provides basic information of NM proteins in wheat and its roles of response to fungal stress.

## Results

### Identification of SUN domain-containing protein candidates

SUN proteins are evolutionarily conserved and appear throughout the animal and plant kingdoms, indicating that SUN domain-containing proteins play essential roles in most organisms (Poulet et al. 2017). Previously, a SUN2-like annotated protein showed the highest connectivity with differentially accumulated proteins (DAPs) in wheat response to fungal stress (Zhang et al. 2019). To further clarify the functional roles of SUN in wheat, we identified SUN domain-containing proteins through BLAST homology searches of the wheat proteome using the Sad1/UNC-84 domain (PF07738) as the reference sequence. After removing the protein sequences with *E*-values > 0.0001 or *n*-values > 1, we identified 4 proteins encoded by 12 genes (Table S1). According to the naming convention and hexaploid characterization of wheat, we named the SUN domain-containing proteins as TaSUN1–4. Like other plant species, SUN proteins of wheat can be divided into two sub-families, Cter-SUN and mid-SUN proteins, based on the conserved domains and transmembrane topology.

**Fig. 1:**
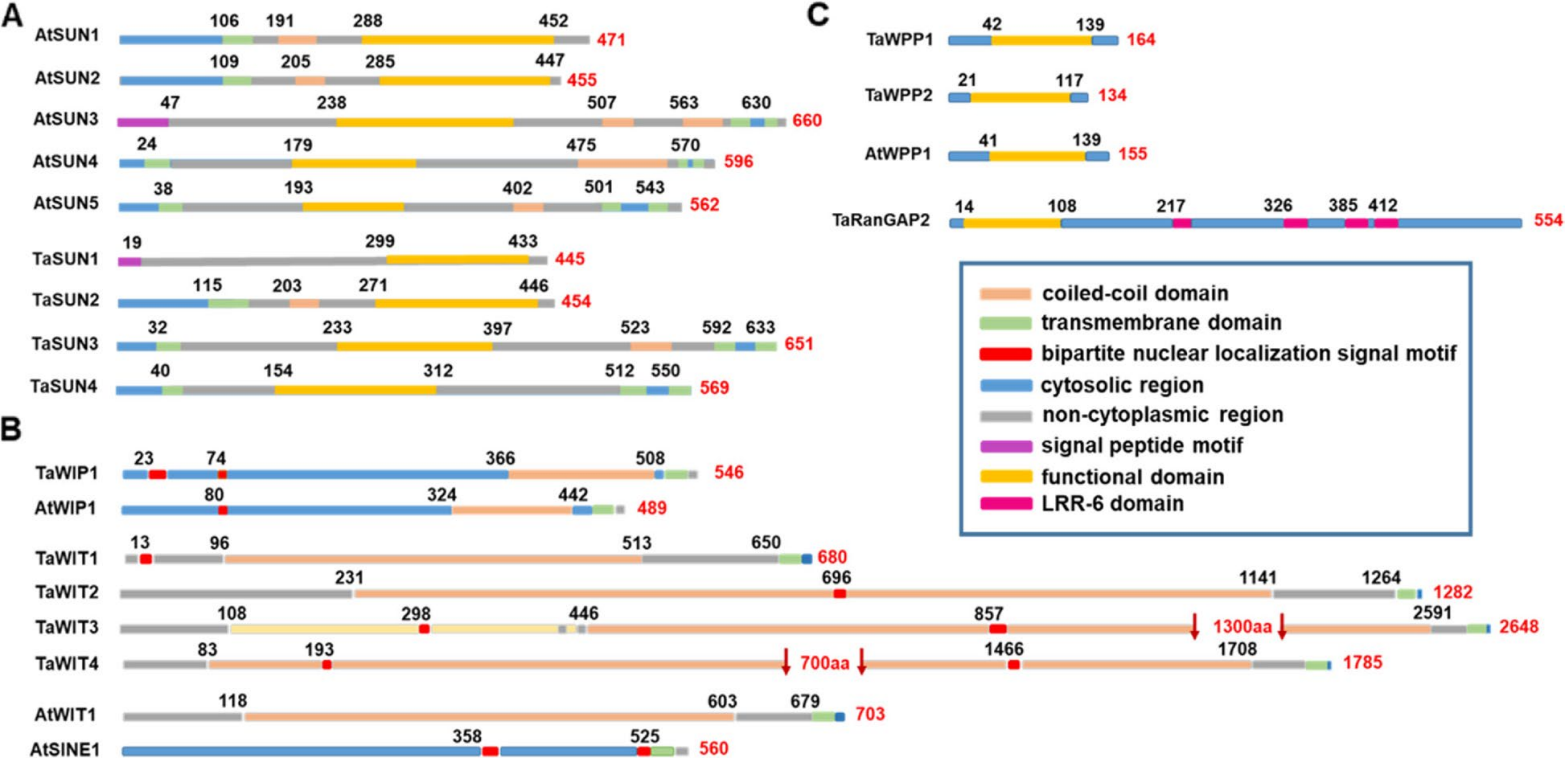
Sequence of SUN and KASH proteins. **A** Comparison of four SUN domain-containing proteins with AtSUNs. According to the location of the SUN-domain (yellow), TaSUN1 and TaSUN2 are assigned to Cter-SUN, while TaSUN3 and TaSUN4 are assigned to mid-SUN. TaSUN1 contains a signal peptide (purple), and the whole sequence is located in the non-cytoplasmic region (gray). Like Arabidopsis Cter-SUNs, TaSUN2 contains a transmembrane domain (green) a coiled-coil (orange), and a N-terminal in the cytoplasmic region (blue). The structures of mid-SUNs are basically the same, with a SUN-domain in the middle, and three transmembrane domains contained within the whole sequence. **B** Five novel coiled-coil transmembrane domain proteins interact with WPP domain-containing proteins. The domain structures of the WIT and WIP protein families are similar. The domain structure is characterized by the presence of an extended coiled-coil domain (orange) and a single C-terminal transmembrane domain (green). The AtWIT1 protein does not contain a bipartite nuclear localization signal motif (red), while the coiled-coil domain has not been identified in the SINE protein sequences. The WIP and SINE protein family members contained a predicted cytosolic N-terminal region (blue) and the C-terminal tail in the non-cytoplasmic region (grey), whereas the opposite pattern is observed in the WIT protein family members. TaWIT3 and TaWIT4 are devoid of 1,300 aa and 700 aa, respectively, in the middle of the coiled-coil domains. **C** Two novel WPP domain-containing proteins. The WPP domain structure is marked in yellow and is characterized by the presence of WPPXXXTR motif. Compared with RanGAP2-like, the WPP proteins are devoid of a C-terminal fragment of the LRR-6 domain (pink) repeat region

The sequence structure and length of TaSUN2 were consistent with this of Cter-SUN of Arabidopsis in that cytoplasmic N-terminal and SUN-domain located in the non-cytoplasmic C-terminal were linked by a transmembrane domain and a coiled-coil domain (Fig. 1A). Interestingly, although the SUN domain of TaSUN1 was also located at the C-terminal, the transmembrane domain was replaced by a N-terminal signal peptide, leading to the non-cytoplasmic localization of the whole sequence. In addition, two mid-SUN structural proteins, TaSUN3 and TaSUN4, identified in wheat showed structural similarities with AtSUN4 and AtSUN5. The mid-SUN proteins were characterized by the existence of three transmembrane domains adjacent to N- and C-terminals, while the SUN domain exists in the middle of the protein, and were distinguished from the TaSUN3 that TaSUN4 protein sequences by the absence of a coiled-coil domain. Notably, TaSUN1 appeared to be a truncated or recombinant form of Cter-SUN and mid-SUN, containing the sole signal peptide of TaSUNs. Furthermore, the SUN domain was always contained in the non-cytoplasmic region in all reported SUN proteins, indicating that the SUN domain may plays an important role as a membrane-bound protein (Fig. 1A).

### Identification of SUN domain-interacting NE protein candidates

With the exception of the coiled-coil domain and the C-terminal transmembrane domain, the KASH domains of plant WIPs share little similarity in terms of amino acids (aa) (Xu et al. 2007; Zhou et al. 2014). However, the KASH domains of opisthokont KASH proteins share a C-terminal 4-aa motif with AtWIPs (including SINEs), which is critical for interacting with the SUN domain and for NE localization (Zhou et al. 2015a; Yang et al. 2017). Thus, we investigated the KASH proteins involved in response to fungal infection using a method described by Zhou (Zhou et al. 2014). We first identified protein sequences with a putative KASH domain in DAPs generated from fungi infected wheat leaves (PeptideAtlas: PASS00682 and PASS00999). We then filtered the sequences based on homology *E*-values < 0.0001 using BlastP in the NCBI database. Using XXPT (X represents any amino acid) as the C-terminal 4-aa pattern and coupled with the SMC_N superfamily domain (PTHR34562), we identified two putative KASH candidates: TraesCS5B02G275100.1 and TraesCS5B02G256500.1. However, the C-terminal of a transmembrane domain (TMD) was detected only in TraesCS5B02G256500.1 using TMpred software (https://embnet.vital-it.ch/software/TMPRED). After confirming its homology with the WPP domain-interacting protein in *Aegilops tauschii* by BlastP alignment, we named this protein TaWIP1. InterPro protein analysis showed the structural similarity of TaWIP1 with AtSINE1, with the exception of the presence of the coiled-coil TMD as shown in Fig. 1B. The N-terminal sequences of WIP proteins functioned in the cytoplasm and were followed by a transmembrane helix (TMhelix), while only the C-terminal sequences were predicted to form part of the non-cytoplasmic domain.

The AtWITs were first identified by tandem affinity purification (Zhao et al. 2008), and showed similar domain organization to the WIP protein family, with a combination of coiled-coil and transmembrane domains (CC-TMD), although no external amino acid similarity was detected between the two protein families. Analysis of the primary structure of WITs of several plant species using Bioedit software revealed the presence of a loose MLE-X(n)-LEK pattern in WITs. Using this pattern, we screened out 58 proteins from the proteome database generated from pathogen-inoculated resistance wheat leaves. Furthermore, four WIT homolog protein candidates were identified by analyzing the C-terminal TMhelix: TraesCS7D02G038700.1, TraesCS6A02G212800.2, TraesCS5B02G074200.1 and TraesCS2A02G421100.1. BlastP alignment showed a high degree of homology of the amino acid sequence of TraesCS7D02G038700.1 with that of WPP domain-interacting tail-anchored protein 1 (WIT1)-like isoform of *A. tauschii*, while the other WIT homolog protein candidates were annotated to the nucleoprotein TPR (first described as translocated promoter region) isoform of *Brachypodium distachyon*, kinesin-like protein and myosin-2 heavy chain-like protein of *A. tauschii* with identities of 83%, 97% and 96%, respectively. The amino acid sequence of myosin-2 heavy chain-like protein showed distinct differences compared to myosin-2 protein of *A. thaliana* (accession number: AED95023.1), such as the loss of two transmembrane domains and plant myosin motor domain (class XI) (Fig. S1). Similarly, the nucleoprotein TPR isoform lost the signal peptide, five transmembrane domains and nucleoprotein TPR-related domain of nucleoprotein TPR (accession EMT01138.1). The kinesin-like protein isoform encoded by TraesCS5B02G074200.1 gained a C-terminal TMhelix due to a change and deletion in C-terminal of Kinesin-like protein (XP_020192443.1). Thus, these proteins differed structurally from the annotated homolog proteins and were hence named TaWIT1 (TraesCS7D02G038700.1), TaWIT2 (TraesCS6A02G212800.2), TaWIT3 (TraesCS5B02G074200.1) and TaWIT4 (TraesCS2A02G421100.1) for the convenience of description. Based on the definition and the position of the predicted transmembrane domain, both WIP and WIT proteins were further classified as tail-anchored (TA) proteins (Borgese and Fasana 2011), consisting of CC-TMD (Fig. 1B). However, WIT protein family members were characterized by a cytoplasmic C-terminal region, while WIP proteins contained a cytosolic N-terminal region. A bipartite nuclear localization signal motif (NLS) was observed in TaWIT1, but not in AtWIT1.

### Identification of WPP domain-containing NE-associated protein candidates

MFP1 attachment factor 1 (MAF1) and its homologs, WPP1 and WPP2, are plant-specific NE-associated protein first identified in *Lycopersicon esculentum* and *A. thaliana* (Patel et al. 2004). Similarly, alignment of MAF1, RanGAPs and AtWPPs showed that the conserved WPP domain was characterized by the WPPXXX[TS]R pattern. Using this pattern, we identified three WPP domain-containing proteins in wheat responding to fungi, encoded by TraesCS2B02G210200.1, TraesCS4A02G051500.1 (names TaWPP1 and TaWPP2, respectively) and TraesCS3B02G433100.1. The TraesCS3B02G433100.1 protein was homologous to RanGAP2-like protein. As shown in Fig. 1C, the WPP proteins are leucine-rich repeat (LRR) region-truncated versions of the RanGAP2-like protein.

### TaSUN1, TaSUN2, TaWIP1, TaWIT1 and TaRanGAP2 are localized to the plant NE

**Fig. 2:**
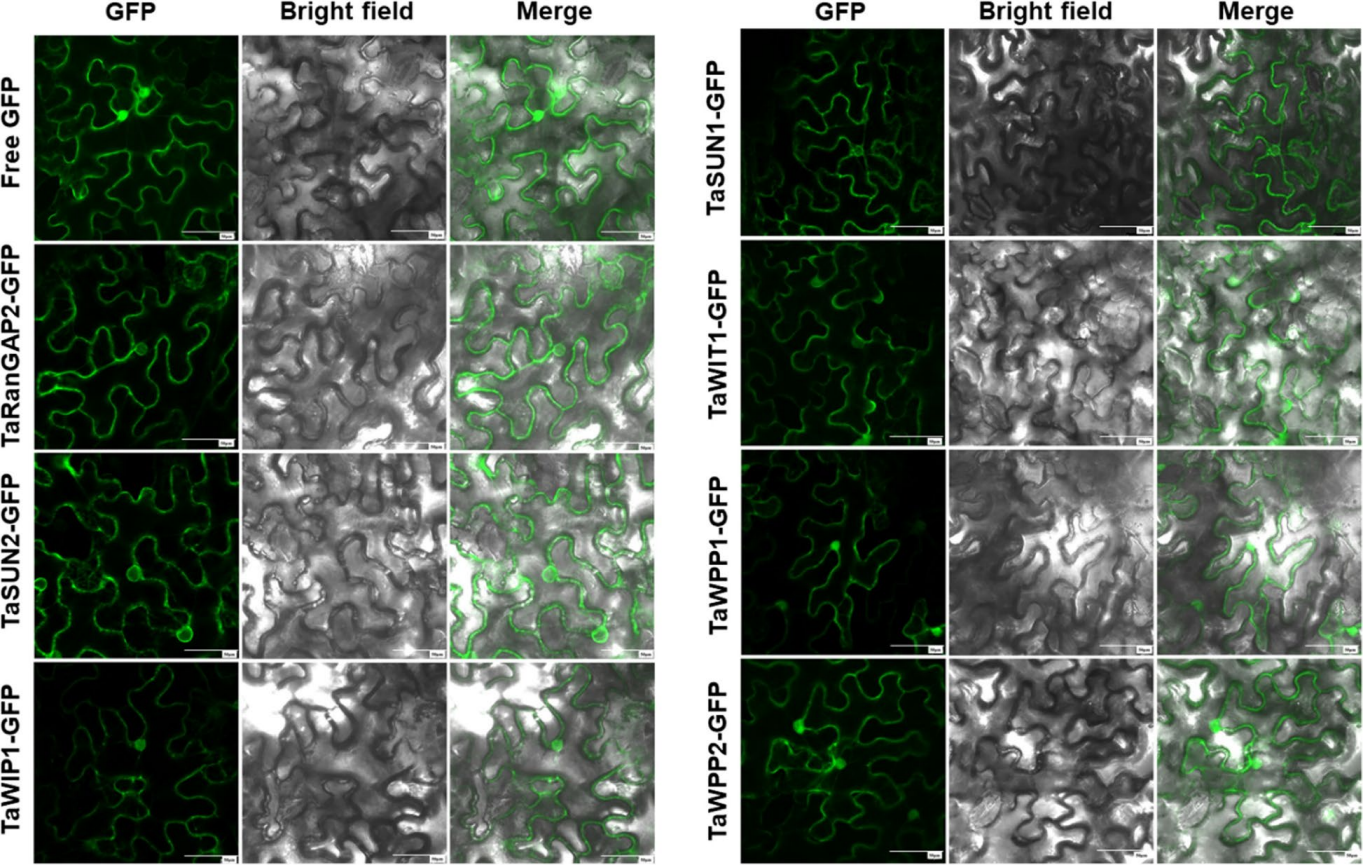
Transient expression and localization of SUN and KASH proteins. TaSUN1, TaSUN2, TaWIP1, TaWIT1 and TaRanGAP2 localize to the nuclear envelope in *N. benthamiana* leaf epidermal cells, while TaWPP1 and TaWPP2 localize like free GFP. Free GFP, TaSUN and putative SUN-interacting nuclear envelope proteins fusion construct under the control of the CaMV-35S promoter were transiently expressed into *N. benthamiana* epidermal cells. Scale bar, 10 µm

KASH proteins are located in the NE and interact directly with SUN proteins. To determine the subcellular localization of these predicted wheat SUN and KASH candidates, vectors were constructed to transiently express C-terminally green fluorescent protein (GFP)-tagged proteins under the control of 35S promoter. Three days after agroinfiltration of *Nicotiana benthamiana* leaves, five fusion proteins, TaRanGAP2, TaSUN1, TaSUN2, TaWIP1, TaWIT1, were detected as circular signals related to the cell membrane and NE (Fig. 2). The NE localization of these proteins provides evidence that these could be NM proteins, and supports the possibility of their interaction to form a complex in NE. Since TaWPP1 and TaWPP2 are characterized with truncated RanGAP2-like, we also tested their subcellular localization. Like free GFP, TaWPP1-GFP and TaWPP2-GFP were located in the nucleus and cytoplasm. Compared with the location of TaRanGAP2, this indicated that the NE localization of WPP domain protein depends on the integrity of WPP and the subsequent domains, although the mechanism is unclear.

### LINC complexes are formed by the interaction of TaWIP1, TaWIT1, TaWPPs and TaSUNs

**Fig. 3:**
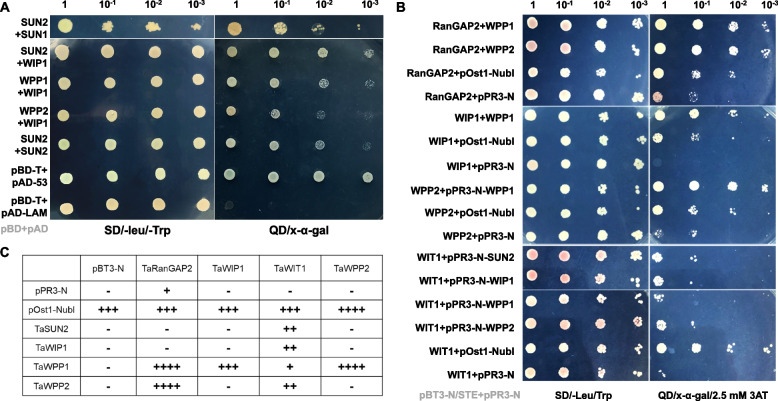
Interactions between the SUN and KASH proteins in the two-hybrid systems. **A** Interactions between TaSUN1, TaSUN2, TaWIP1, TaWPP1 and TaWPP2 detected in the nuclear two-hybrid system. pGBKT7-53 and pGBKT7-LAM were used as positive and negative controls, respectively. **B** MYTH assays between the TaRanGAP2, TaWIP1, TaWIT1 and TaWPP2 as bait and TaSUN2, TaWIP1, TaWPP1 and TaWPP2 as prey. pOst1-NubI and pPR3-N were used as positive control and negative controls, respectively, after co-transfection with the target gene. Spots corresponding to 100 000 cells on permissive medium (PM) and test medium (TM) are shown. **C** Tabulated summaries of the strength of protein interactions established based on growth efficiency on solid TM. SD/-Leu/-Trp, double-dropout medium without leucine and tryptophan; QD/x-α-gal, quadruple dropout medium with x-α-gal

**Fig. 4:**
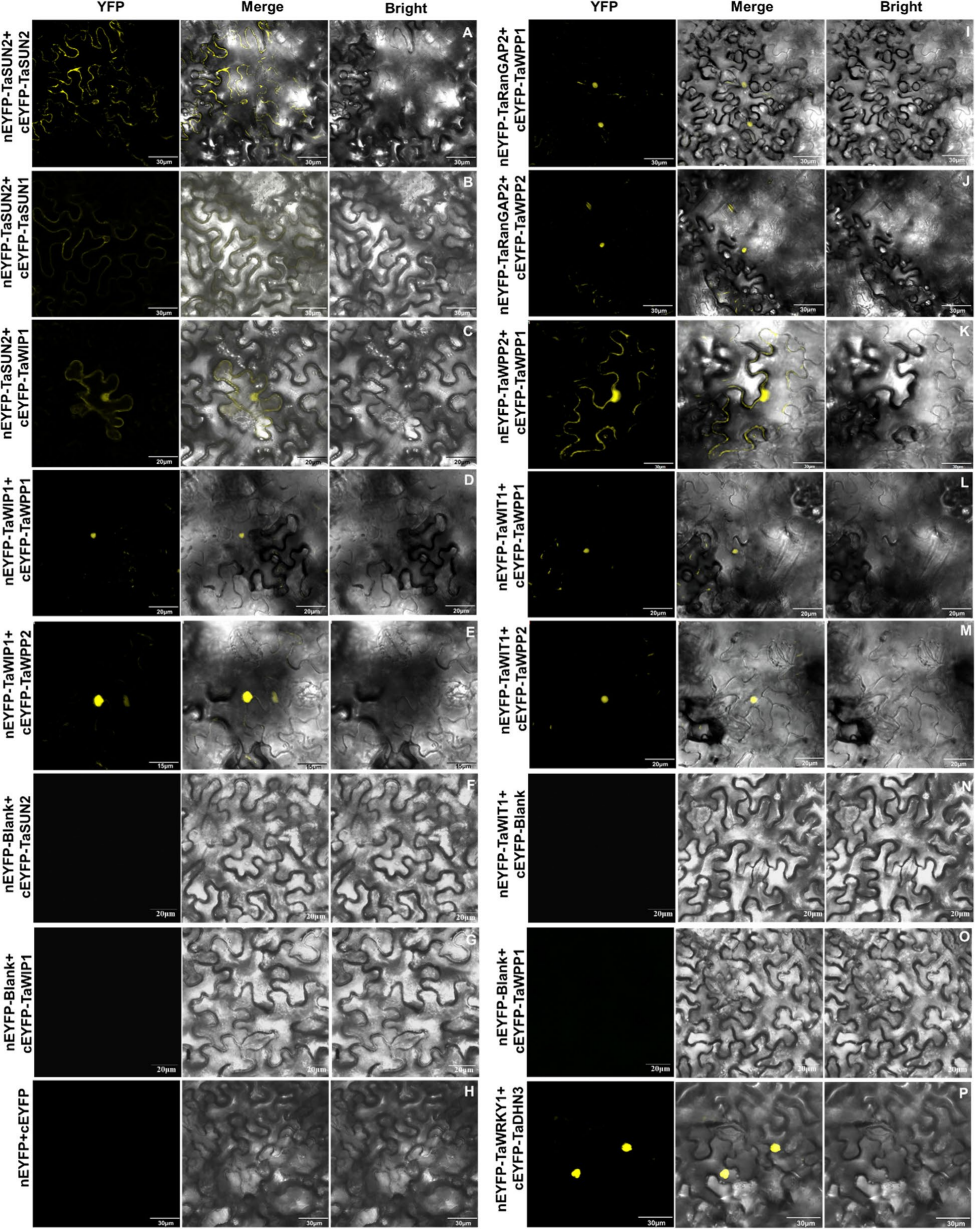
Interactions between the SUN and KASH proteins in the bimolecular fluorescence complementation (BiFC) assays. The SUN and quasi-interacting protein coding sequences were cloned into nEYFP and cEYFP. After transiently co-transforming *N. benthamiana* leaf epidermal cells with the nEYFP and cEYFP recombinant vectors, YFP fluorescence confocal micrographs show the location of the interaction. nEYFP and cEYFP empty vectors were co-transfected in *N. benthamiana* as a negative control, while nEYFP-TaWRKY1-2D and cEYFP-TaDHN3 were employed as a positive control. Scale bars are shown in the lower right corner of each image

**Fig. 5:**
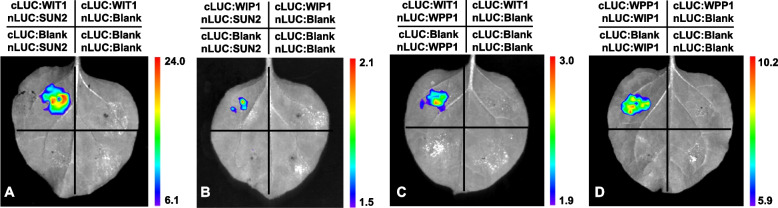
The activity of TaWIP1, TaWIT1 interacting with both TaSUN2 and TaWPP1 detected by luciferase complementation assay. Photographs of representative leaves were taken 2 d after agroinfiltration. The right pseudocolor bar shows the range of luminescence intensity (e + 05) in each image from high (red) to low (blue)

To address the specificity of the TaWIP1, TaWITs, TaWPPs and SUN-like protein interactions, we evaluated the affinity of WIP1 for WIT, WPP protein family members and SUN-like using a yeast two-hybrid system, luciferase complementation (LUC) and bimolecular fluorescence complementation (BiFC) assays. Since some LINC proteins are not localized in the nuclear membrane, we used both the nuclear yeast two-hybrid (Y2H) system and the split-ubiquitin-based membrane yeast two-hybrid (MYTH) system to test all interactions. In the nuclear Y2H system, we cloned all the protein encoding sequences under investigation into pGBKT7 and detected their self-activation activity in triple dropout medium (TDO/x-α-gal; SD/-Trp/-His/-Ade/x-α-gal). Among these genes, only TaWIP1 grew in triple dropout medium and exhibited self-activation activity (Fig. S2A). Subsequent analysis of the interactions between SUN and KASH proteins indicated that TaSUN2 interacts not only with itself to form homologous polymers (Fig. 3A), but also with TaSUN1 to form a heterologous polymer, and with TaWIP1 (Fig. 3A), hinting a form of SUN-KASH NE bridge. These interactions were confirmed by BiFC assays, which also revealed their location in the nuclear membrane (Fig. 4A, B and C). Moreover, TaWIP1 showed significant interaction with TaWPP1 and TaWPP2 in the nucleus (Figs. 3A, 4D and E). Furthermore, after we examined the expression and self-activation of these proteins (Fig. S2B), we also detected their interactions using the MYTH system (Fig. 3B and C). BiFC assays and the growth of yeast colonies on QDO indicated that TaRanGAP2 had strong interactions with both TaWPP1 and TaWPP2 in nucleus (Figs. 3B; 4I and J), while TaWPP1 and TaWPP2 also strongly interacted with each other to form heterodimers without self-interaction in both the nucleus and cell membrane (Figs. 3B; 4K). Intriguingly, we detected a moderate interaction between TaWIT1 and TaSUN2 (Figs. 3B; 5A). Since both the WIT and WIP protein families contain predicted coiled-coil domains with possible dimerization ability, we also evaluated the interaction between TaWIT1 and TaWIP1 protein (Fig. 3B). YFP fluorescence confocal micrographs indicated that TaWIT1 also interacted with TaWPP1 and TaWPP2 in the nucleus (Fig. 4L and M). Additionally, these interaction relationships were substantiated by LUC assay similarly (Fig. 5). These findings provided evidence that the KASH proteins TaWIT1/TaWIP1 and TaSUN2 form a LINC complex connecting the inner and outer nuclear membranes. At the same time, the plant-specific small proteins TaWPP1 and TaWPP2 also physically linked TaWIT1/TaWIP1 to TaRanGAP2. Based on our results and previous reports, we hypothesized the existence of a NE complex composed of SUN-WIP-WIT-WPP in wheat.

### TaSUN2 regulates the expression of defense-related genes to mediate innate immunity to fungi

It has been reported that SINE2 contributes to innate immunity against an oomycete pathogen (Zhou et al. 2014) and TaSUN2 is involved into the response of wheat to fungal stress (Zhang et al. 2019); we therefore investigated their function in disease defense. First, we measured the expression levels of *TaSUN2* and KASH protein encoding genes in wheat near-isogenic lines (NILs) N9134R/N9134S and 1013R/1013S that exhibited resistance or susceptibility to infection by *Bgt* and *Pst*, respectively. RT-qPCR analysis indicated that *TaRanGAP2* was significantly upregulated in the powdery mildew resistant near-isogenic line 9134R/S at 6 hpi. *TaSUN2* and *TaWPP1* were also significantly upregulated in 9134R at 24 hpi, while weaker upregulation was observed in 9134S. *TaRanGAP2* and *TaSUN2* were also upregulated in the near-isogenic line 1013R/S following stripe rust infection (Fig. S3).

**Fig. 6:**
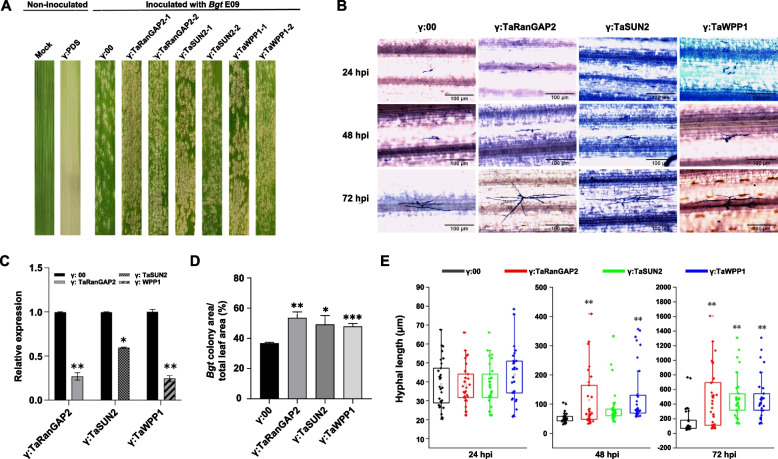
Silencing of *TaRanGAP2*, *TaSUN2*, or *TaWPP1* reduced resistance to powdery mildew. **A** Photobleaching of wheat seedlings (four-leaf stage) leaves at 10 dpi with BSMV-PDS; disease phenotype in wheat seedlings (four-leaf stage) leaves inoculated with *Bgt* E09 at 10 dpi with BSMV-γ. **B** Representative microscopic images of single colonies of powdery mildew on leaves with monogenic silencing. **C** RT-qPCR confirmation of decreased expression of TaRanGAP2, TaSUN2, and TaWPP3 in leaves with monogenic silencing. **D** Colony area of *Bgt*-infected leaves; data represent the mean ± SD (*n* = 9 leaves) from three separate experiments. **E** The average number of total hyphal length of each colony counted on leaves sampled at 24, 48, and 72 hpi of each genotype; data represent the mean ± SD (≥ 50 colonies per genotype) from three independent. **, *P* < 0.01 (Student’s *t*-test)

**Fig. 7:**
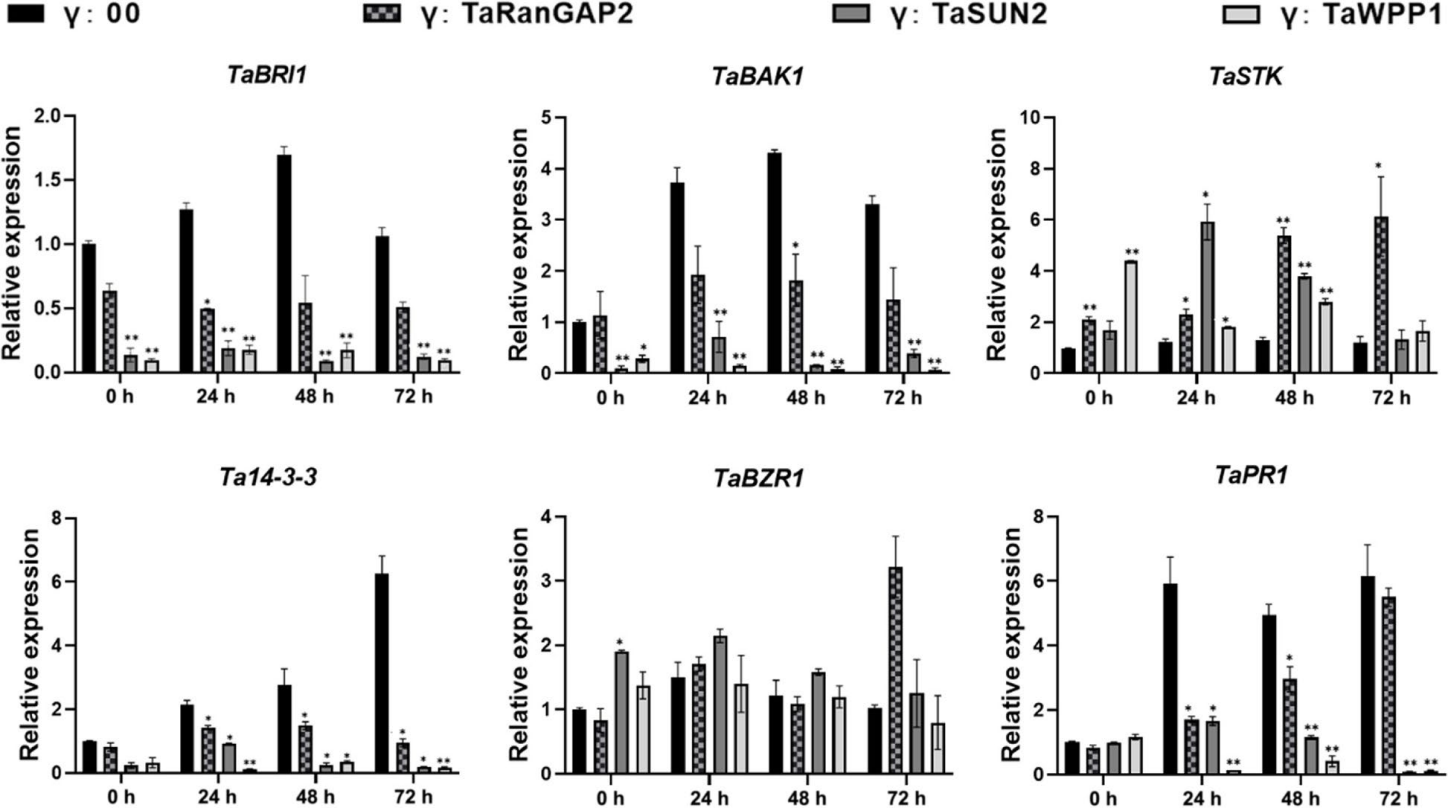
Relative transcript levels of *TaBRI1*, *TaBAK1*, *TaSTK*, *Ta14-3–3*, *TaBZR1*, and *TaPR1* in *TaRanGAP2*-, *TaSUN2*- or *TaWPP1*-slienced plants inoculated with *Bgt*. Relative gene expression of the silenced line inoculated with *Bgt* compared with that in control plants at 0 h using *Actin* as the internal reference. Data represent means ± standard errors (*n* = 3 biological replicates) of three independent experiments. Differences between time-course sampling points were assessed using SSPS. *P < 0.05

To further evaluate their roles in the stress response to fungal infection, we knocked down the expression of *TaRanGAP2*, *TaSUN2* and *TaWPP1* using the BSMV RNA-induced gene silencing (VIGS) system in the near isogenic lines N9134R/S. This pair of NILs is only different in a powdery mildew resistance loci *PmAS846*. By 10 days post-inoculation (dpi), the chlorotic phenotype with obvious photobleaching was observed on the third and fourth leaves of 9134R/S inoculated with BSMV-γ-PDS, suggesting high-efficiency silencing. *Bgt* E09 was then evenly inoculated on the fourth leaf of BSMV-target pre-inoculated 9134R/S. After 9 dpi, the powdery mildew colonization of the fourth leaf of *TaRanGAP2*-, *TaSUN2*-, *TaWPP1*-silenced plants were significantly greater than that in BSMV-γ0 treated N9134S (Fig. 6A, D). RT-qPCR analysis showed that the expression of *TaRanGAP2*, *TaSUN2*, and *TaWPP1* was significantly inhibited in the silenced plants, with silencing efficiency between 40 and 70% (Fig. 6C). Conversely, the percentage area of fungal colonization on the leaf surface increased significantly, indicating decreased immunity to the powdery mildew pathogen in the *TaRanGAP2*-, *TaSUN2*-, *TaWPP1*-silenced plants (Fig. 6D). In addition, we evaluated the effect of gene silencing on mycelial development of *Bgt*, and found that colony size and development were promoted to different degrees in the three gene-silenced lines (Fig. 6B). At 24 hpi, the mycelium development and length in the *TaRanGAP2*-, *TaSUN2*-, *TaWPP1*-silenced plants was significantly better than that in the control group (Fig. 6E). At 9 dpi with powdery mildew pathogen, there were no obvious changes in N9134R, although speckled flora appeared in half of the gene-silenced plants by 15 dpi (Fig. S4). It has been shown that fungal resistance is accompanied by the response of marker genes, such as *PR1* and *BAK1*. To clarify the relationship between these three genes and downstream defense-related genes, we detected the expression of several stress-related and/or disease resistance-related marker genes. Compared with the control group, the expression levels of *TaBRI1*, *TaBAK1, Ta14-3–3* and *TaPR1* in *TaRanGAP2*-, *TaSUN2*-, *TaWPP1*-silenced plants were markedly decreased, while the expression of *TaSTK* was upregulated 3–6 fold (Fig. 7). These results indicated that TaRanGAP2, TaSUN2 and TaWPP1 play positive roles in wheat resistance to fungal infection by regulating innate immunity.

### Possible functional model of the SUN/KASH complex

**Fig. 8:**
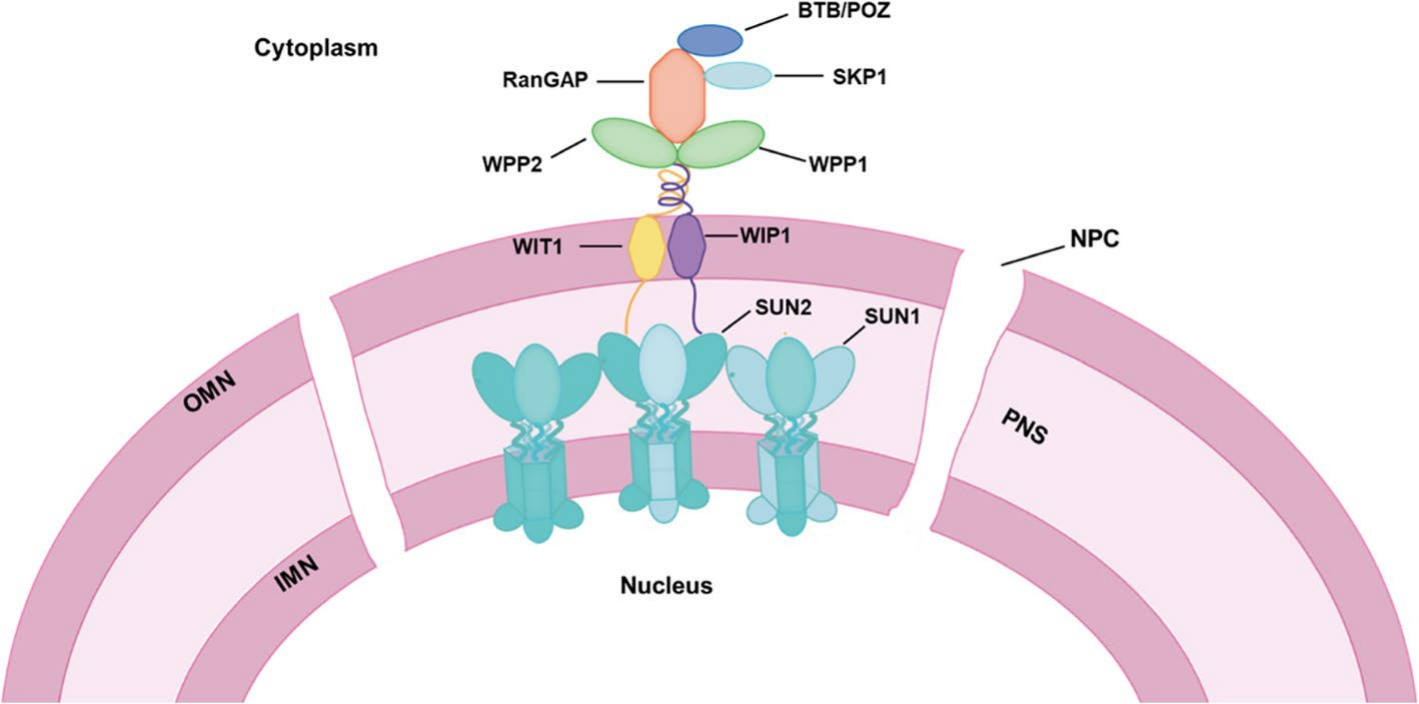
Schematic diagram of LINC complex structure. The C-terminal SUN protein in the form of a homotrimer or heterotrimer located in the inner nuclear membrane interacts with WIP and WIT proteins anchored in the outer nuclear membrane to form a heteropolymer. The proteins containing the WPP domain interact with each other, and both interact simultaneously with TaWIT1 and TaWIP1 to anchor the complex to the nuclear membrane. TaRanGAP2 interacts not only with WPP protein, but also with the disease resistance-related proteins BTB/POZ and SKP1

Plants combat fungal stress by regulating the entry of molecules required to activate defense-related genes into the nucleus, thereby preventing fungal colonization. During this process, NE bridges facilitate the mechano-transduction of signaling molecules and transcripts but not soldier. To further elucidate the resistance-related signaling molecules and the complete structure of the NE complex, we screened a fungi pre-treated Y2H library using TaSUN2 and TaRanGAP2 as bait. The results showed that TaSUN2 not only forms a homotrimer, but also forms a heteropolymer with TaSUN1 (Fig. 3B). Interestingly, among the possible proteins that interact with TaRanGAP2, we found some disease resistance-related proteins, such as zinc finger CCCH domain-containing protein 18-like, TAZ domain-containing protein 2-like and CBL-interacting protein kinase 17-like. In particular, two ubiquitination-related proteins, TaSKP1 and RNF5-like, were also found to interact directly with RanGAP2 (Table S2). The crystal structure of the human SUN and KASH interaction has been determined, and both SUN1 and SUN2 combine to form trimers in vitro, Therefore, based on nuclear membrane and protein structure, we proposed a hypothetical functional model of the SUN/KASH complex in wheat (Fig. 8). In this model, the SUN-KASH complex is recruited or mediates defense-related protein shuttling between the nucleus and cytoplasm by coupling with WPP-RanGAP and activating ubiquitination.

## Discussion

### SUN proteins in plants

Many SUN and LINC complexes have been identified in eukaryotes, particularly in model plants (Graumann et al. 2010; Tamura et al. 2010; Shah et al. 2019). However, because transmembrane nucleoporins are not evolutionarily conserved (Mans et al. 2004), functional analogs of transmembrane nucleoporins have not been identified in wheat. As a nuclear membrane protein, TaSUN2, has high connectivity in the proteome of wheat infected by powdery mildew and stripe rust (Zhang et al. 2019). Here, we identified the SUN protein and the candidate KASH protein in wheat as a hexaploid species with a gigantic genome that encodes gene families with hundreds of members. However, we identified only four SUN proteins encoded by 12 genome nucleotide sequences in 1 and 3 homologous group. Similarly, only five SUN proteins exist in Arabidopsis, five in *Zea mays*, three in *Oryza sativa*, five in *Sorghum bicolor* and four in humans (Tzur et al. 2006; Poulet et al. 2017; Yuan et al. 2021). The genomes of these species are differ greatly, although this is not directly reflected in the number of SUN proteins encoded. Additionally, the simultaneous appearance of Cter-SUN and mid-SUN in unicellular organisms strongly indicates that their evolutionary branches appeared before the evolution of multicellular organisms (Poulet et al. 2017). This confirmed that SUN proteins are evolutionarily conserved in animals and plants (Graumann et al. 2014; Yuan et al. 2021), indicating that SUN proteins are essential for diverse trait performance. SUN transcription levels are also subject to various external influences, such as chemical, hormonal and abiotic stresses. Here, we demonstrated that the expression of *TaSUN2* was induced by both *Bgt* and *Pst* infection (Fig. S3). Shah reported the existence of other stress and pathogen response elements in the SUN promoter, including MBS and HSE (Shah et al. 2019). In addition, the conserved C-terminal SUN domain is in the PNS, and crystallographic analysis indicates that SUN proteins form homotrimers as well as SUN-SUN heterotrimers (Hieda 2017). In this study, we evidenced that TaSUN2 forms homogeneous complexes with itself and heterogeneous complexes with TaSUN1, indicating that the functions of these proteins are diverse and dynamic. A detailed functional dissection of the NE complex will contribute to a better understanding of the mechanism underlying antagonistic traits, such as pathogen resistance and growth, and further promote crop improvement.

### Functional diversity of KASH proteins in plants

To date, the reported functions of WIP and WIT include RanGAP and WPP protein anchors, mediating nuclear movement and nuclear morphology and participating in meiosis (Zhou et al. 2015a). AtSINEs were identified as plant-specific KASH proteins and interacted with SUN1 and SUN2 via the KASH and SUN domains (Zhou et al. 2012a, 2014). In this study, we identified TaWIP1 as a NE protein with a similar structure to that of AtSINEs (Fig. 1B), but divergent sequences compared with the homologous proteins in Arabidopsis. In particular, the putative KASH domain comprises the sequence PSPPEFVPT in TaWIP1 but is QDDDVGYYTVPT in AtSINE1. However, TaWIP1 strongly interacted with TaSUN2 in the same way that AtSINE1 interacts with AtSUN2, indicating that the C-terminal 3-aa (VPT) of KASH are important for SUN and KASH binding. The small C-terminal KASH domain of WIP1 is in the PNS and its long N-terminal domain is in the cytoplasm, whereas as another type of KASH protein, WITs have the opposite sequence structure with a small cytoplasmic-located C-terminal and long non-cytoplasmic N-terminal. Intriguingly, here, we identified four WIT proteins in wheat, and detected moderate interaction between TaSUN2 with TaWIT1, as well as an interaction between TaWIT1 with TaWIP1 in MYTH system, although these interactions were not observed in nuclear Y2H system. This demonstrated that the binding of TaSUN2-TaWIT1 and WIT1-WIP1 depend on the transmembrane localization at the nuclear membrane. In model plants, different KASH proteins, such as SINE and TIK, are specifically involved in root and stomatal complex development, meiotic chromosome separation, guard cell nuclear anchoring and plant immunity (Zhou et al. 2014; Groves et al. 2020). Although the functional variations that result from this difference are not clear, our findings indicated that the functions of KASH protein are diverse, and also supplied additional evidence for functional specialization of different traits in plants.

### Nucleocytoplasmic shuttling of proteins may depend on the RanGAP‑WPP complex

Ras-related nuclear protein (Ran), which is exclusively involved in nucleocytoplasmic transport and localization of plant small GTPase in the NE (Ma et al. 2007), is regulated by guanine nucleotide exchange factors (GEFs) and GTPase accelerating proteins (GAPs) (Fehér and Lajkó 2015). In this study, we identified TaRanGAP2, TaWPP1 and TaWPP2 as WPP domain-containing proteins in wheat after inoculation with fungus, and demonstrated their involvement in wheat defense against the powdery mildew pathogen as a positive regulator similar to TaSUN2. Compared with WPP proteins, TaRanGAP2 is rich in LRR domains, which are commonly found in both receptor and receptor-like proteins that recognize biological stimuli, and are predicted to be the Ran-binding site in AtRanGAP1 (Rodrigo-Peiris et al. 2011; Boruc et al. 2015). RanGAP located around the NPC rapidly captures shuttling Ran-bound GTP and induces hydrolysis to produce the Ran-bound GDP required for the next cycle of effector recognition (Jahed et al. 2016). Targeting the nuclear rim and the cell plate through the interaction of RanGAP with the CC domain of Rx leads to the activation of the Rx-dependent defense response, which requires the complete WPP domain (Jeong et al. 2005; Hao et al. 2013; Sobhanian and Sacco 2014). The WPP protein has also been highlighted as a new plant-specific class of co-chaperone, which participates in the membrane delivery/targeting of multiple substrate proteins that bind to the coiled-coil domain (Meier et al. 2010). Recently, we demonstrated that TaRPP13L1-3D confers powdery mildew resistance in wheat and is transferred into the nucleus through its interaction with TaWPP1, but not TaRanGAP2 (Zhang et al. 2022). In the current study, we showed that silencing *WPP1* significantly reduced the expression of disease defense marker genes *TaBRI1*, *TaBAK1* and *TaPR1* in wheat (Fig. 7). Taken present result that RanGAP2 interacted strongly with WPP1 and WPP2 (Fig. 3) together, we guess that shuttling of pathogen defense-related proteins between the nucleus and cytoplasm depends on the RanGAP‑WPP complex. In terms of subcellular localization, we noted that TaWPP1 and TaWPP2 are not anchored on the nuclear membrane (Fig. 2), but instead exhibited a pattern of expression similar to that of the WPP proteins in the leaf epidermis cells of *A. thaliana* and resembling the distribution of free GFP (Patel et al. 2004). However, following agroinfiltration of the meristematic region of root tip cells, the localization of AtWPP1 and AtWPP2 was consistent with that of MAF1, which accumulated in the NE and showed additional staining in the cytoplasm and nucleus (Gindullis et al. 1999; Patel et al. 2004). Considering that both TaWPP1 and TaWPP2 interact with NE-localized TaWIP1, we hypothesized that SUN-WIP enhances anchoring of WPP domain proteins to the NE, and further formed the SUN-WIP-WPP-RanGAP complex. Thus, WPP proteins may function as an important cofactor for recruiting and anchoring defense-related proteins to NE, while SUN-WIP/WIT acts as a transmission bridge to transmit proteins or molecular signals into the nucleus.

### The potential NE-associated complex assists resistance-related proteins transfer though NE in the response of wheat to fungal infection

The constitutive expresser of pathogenesis-related genes 5 (CPR5) nucleoporin is an inhibitor of PCD and ETI (Gu et al. 2016). To identify a protein with similar function in wheat, we screened a fungi-pretreated yeast library using SUN2 and RanGAP2 as bait. Unfortunately, we did not detect any analogs of CPR5, or similar four-transmembrane structures. However, a large number of candidates interacting with RanGAP2 were screened out, including zinc finger protein and CBL-interacting protein kinase (Table S2). Although the function of zinc finger CCCH domain-containing protein 18 has not been reported, the *Oryza sativa* CCCH-type zinc finger protein, C3H12, has been identified as a nucleic acid-binding protein that positively and quantitatively regulates rice resistance to *Xanthomonas oryzae* pv *oryzae* likely via the JA-dependent pathway (Deng et al. 2012). The tandem CCCH zinc finger protein, CaC3H14, is transcriptionally targeted by CaWRKY40 and enhances the defense response of pepper plants to infection by *R. solanacearum* (Qiu et al. 2018). In contrast, the C2H2 zinc finger protein is predominantly associated with disease resistance in plants and participates in abscisic acid (ABA)-induced plant antioxidant defense (Huang et al. 2009; Sun et al. 2010; Guan et al. 2014). In addition, CBL-interacting protein kinase plays an important role in adapting to stress conditions (Sardar et al. 2017; Lin et al. 2021; Xu et al. 2021). CBL-interacting protein kinase (CIPK) is required for biotic stress tolerance of plants in plant-pathogen interactions (Liu et al. 2019). The TaCBL4-TaCIPK5 complex positively modulates wheat resistance to *Pst* in a ROS-dependent manner (Liu et al. 2018); TaCIPK10 interacts with and phosphorylates TaNH2 to regulate wheat resistance to *Pst* (Liu et al. 2019). CBL2-CIPK31-AKT1L is a newly identified calcium signaling pathway that positively regulates rice defense against disease (Lin et al. 2021). Although the interaction and function remain to be elucidated, the identification of this protein provides a platform for identifying further SUN-KASH-dependent resistance proteins. Furthermore, our results raise the possibility that the functions of the NE complex in the responses of plants to fungal challenge are controlled by the expression level of each component. In conclusion, these insights into the function of the SUN-WIP/WIT-WPP-RanGAP complex will be useful in clarifying the mechanism of stripe rust and powdery mildew resistance in wheat, and enriching our knowledge of the signals transmitted during pathogen defense responses.

## Materials and methods

### Plant materials and fungal isolates

The winter wheat line N9134, which has maintained a high level of resistance to all powdery mildew pathogens, was backcrossed with the susceptible material Shaanyou 225 seven times and followed by self-crossed twice to obtain a near-isogenic line N9134R2 and N9134S2 with resistance and susceptibility to powdery mildew (Guo et al. 2021). The *Bgt* isolate E09 was preserved in vivo on Shaanyou 225 plants. Both N9134R/S and Shaanyou 225 were cultivated at 18 ℃ in a 1:1 mixture of substrate and soil under a 16 h light/8 h dark photoperiod. N9134R/S 7- day-old seedlings were inoculated with fresh *Bgt* E09 conidia. At 0, 24, 48, and hours post-inoculation 72 (hpi), the second leaf of was harvested and immediately placed into liquid nitrogen prior to storage at -80 ℃. All samples are collected from three biological replicates (Zhang et al. 2014).

### Identification of SUN and KASH protein candidates

SUN domain-containing proteins was identified through BLAST homology searches of the wheat proteome using the Sad1/UNC-84 domain (PF07738) motif. KASH proteins were identified from DAPs data that generated from fungi infected wheat leaves (PeptideAtlas: PASS00682 and PASS00999) with the conserved coiled-coil domain and the C-terminal transmembrane domain coupling the C-terminal 4-aa motif as described by Zhou (2014). WIT proteins and WPP domain-containing protein candidates were identified using the developed loose pattern, which were generated by alignment of WITs and WPP homologue sequence of other plant species, respectively.

### RNA extraction and RT-qPCR assays

Total RNA was extracted from the leaves of N9134R/S collected at each time-point using TRIzol reagent (Invitrogen) and further purified with DNase I (TaKaRa, Dalian, China). The first complementary strand cDNA was synthesized using the PrimeScript™ RT reagent kit (perfect real-time) (Zhang et al. 2013). SYBR Green-based real-time quantitative reverse transcription PCR (RT-qPCR) was used to analyze the expression profiles of *TaRanGAP2*, *TaSUN2*, and *TaWPP1* in 1013R/S and N9134R/S leaves after inoculation with *Pst* and *Bgt*, respectively, in three independent technical replicates at designated time-points (0, 6, 12, 24, 36, 48, 72 and 96 h post-inoculation (hpi) for *Bgt*; 0, 24, 48, 72, 96 and 120 hpi for *Pst*). The expression profiles of the stress-related genes *TaBRI1*, *TaBAK2*, *TaSTK*, *Ta14-3–3*, *TaBZR1* and *TaPR1* in the silenced lines infected with powdery mildew at 0, 24, 48 and 72dpi were also analyzed. The experiment relies on QuantStudio™ 7 Flex Real-Time PCR System (Life Technologies Corporation, USA).

### Subcellular localization

The pYJ:GFP vector carrying the strong cauliflower mosaic virus (CaMV) 35S promoter was cleaved with *Spe*I restriction enzyme and forward and reverse primers containing a *Spe*I linker (Table S3) were designed to amplify the open reading frame (ORF) of the target gene using Primestar® HS DNA polymerase (TaKaRa). The target gene with linker was ligated into the linearized vector fragment using the ClonExpress MultiS One Step Cloning Kit (Vazyme) to generate wheat TaRanGAP2-GFP, TaSUN1-GFP, TaSUN2-GFP, TaWIP1-GFP, TaWIT1-GFP, TaWPP1-GFP and TaWPP2-GFP fusion vectors and sequenced to verify normal coding. The recombinant plasmid and control vector pYJ:GFP were then transformed into *Agrobacterium tumefaciens* GV3101 and injected into tobacco plant epidermal cells. After 48 h of culture under normal growth conditions, the leaves were cut off to evaluate the GFP fluorescence signal of the fusion protein under an Olympus fluorescence microscope.

### Yeast 2-hybrid signal sequence trap system

In the yeast 2-hybrid (Y2H) system, we first cleaved pGBKT7 and pGADT7 to generate two linear vector fragments using the restriction endonucleases *EcoR*I and *BamH*I. According to the ORF sequence of the target gene, we designed AD-F/R and BD-F/R linkers containing *EcoR* I and *BamH* I sites (Table S3), and constructed the pGBKT7 and pGADT7 fusion vectors containing the target sequence. The pGBKT7 fusion vector was transfected into Y2H yeast according to the method described by Guo et al. (2021) and self-activation was evaluated by detection of TDO/X-α-gal activity. The pGBKT7 and pGADT7 fusion vectors were then co-transferred into Y2H yeast and the interaction was evaluated by detection of QO/x-a-gal activity. In the split ubiquitin-based MYTH, the target genes were cloned separately into the bait and prey vectors using the linker primers shown in Table S3. The recombinant bait vector was transfected into NMY51 for functional detection and self-activation detection and interaction was evaluated as QO/x-α-gal/2.5 mM 3AT using dual membrane starter kits User Manual (Dual systems Biotech) according to the manufacturer’s instructions. In the membrane system screening library, the yeast library plasmid (10 μg) was transfected into NMY51 containing pBT3-N-TaRanGAP2 recombinant plasmid, and plated into QD/x-a-gal culture dishes. After three days, healthy monoclonal colonies were selected and the plasmid was extracted using TIANprep Yeast Plasmid DNA Kit (TIANGEN). The T7 and 3'AD primers were used for colony PCR, and the inserted cDNA library fragment was obtained and sequenced. The sequencing results were analyzed using NCBI (http://blast.ncbi.nlm.nih.gov/Blast.cgi) and Ensembl plants (https://plants.ensembl.org).

### BSMV-mediated gene silencing

After digestion of BSMV-γ with the *Sap*I restriction enzyme to generate linearized vector fragments, the specific gene fragment (approximately 300 bp) was amplified from the target gene using specific primers containing a *Sap*I linker (Table S3) for generation of the recombinant vector by homologous recombination. Equal amounts of BSMV-γ, BSMV-β and BSMV-α were transfected into competent *Agrobacterium tumefaciens* GV3101 and injected into four-leaf-stage *N. Benthamiana* leaves. The empty γ0 vector was used as a negative control, while BSMV-γ-TaPDS carrying a 214-bp fragment of the wheat phytoene desaturase (PDS) gene was generated as a positive control of the silencing system. At 7 days post-infection, *N. benthamiana* leaves were collected, ground and applied to N9134R/S 2.5-leaf-stage seedlings as described previously (Zhang et al. 2022). After incubation for 7 days at 18 ℃–22 ℃ in a greenhouse, the infected seedling leaves were inoculated with *Bgt* E09 until powdery mildew symptoms appeared. This experiment was repeated three times.

### Bimolecular fluorescence complementation (BiFC) and luciferase complementation (LUC) assay

The coding regions of TaSUNs, TaWIP, TaWIT, TaRanGAP2 and TaWPPs were subcloned into the pn-EYFP and pc-EYFP vectors using the *BstB*I restriction sites to generate the pn/cEYFP-TaSUN1, pn/cEYFP-TaSUN2, pn/cEYFP-TaWIP1, pn/cEYFP-TaWIT1, pn/cEYFP-TaRanGAP2, pn/cEYFP-TaWPP1, and pn/cEYFP-TaWPP2 vectors, respectively. Similarly, these coding genes were subcloned into pLucN-JW771 and/or pLucC-JW772 vectors after digestion by *KpnI* and *SalI* for LUC assay. These constructs were then transiently co-expressed in *N. benthamiana* epidermal cells using the transformation methods described for evaluation of subcellular localization.

### Histological analysis

A section of approximately 1.5–2.0 cm in the middle of N9134R/S leaves were collected at 24, 48 and 72 hpi after powdery mildew inoculation. The leaves were immersed in a mixture of absolute ethanol/glacial acetic acid (3:1; v/v) fixative containing 0.15% (v/v) trichloroacetic acid overnight at room temperature for decolorizing. The leaves were then stained with 0.6% Coomassie brilliant blue G-250 for 24 h. After washing the leaves with ddH_2_O, the leave samples were preserved in glacial acetic acid/glycerin (1:4 v/v) and the development and number of powdery mildew hyphae at each time-point were determined using an Olympus BX61 fluorescent microscope.

## Statistical analysis

GraphPad Prism 9 was used for statistical analysis. Data were presented as the mean ± standard deviation (SD). Differences between groups were evaluated using Student’s *t*-test with a threshold of *P* < 0.05 was set as the threshold for statistical significance. Image J was used to calculate the hyphal development length and the ratio of *Bgt* colony area to leaf area. Origin 2022 and Adobe Illustrator 2020 were also used for mapping.

### Supplementary Information

**Additional file 1: Figure S1.** Comparison of TaWIT2, TaWIT3 and TaWIT4 with the annotated homologs. **Figure S2.** Self-activation detection of SUN and KASH proteins. **Figure S3.** Relative transcript levels of TaRanGAP2, TaSUN2, and TaWPP1 in N9134R/S and 1013R/S inoculated with Bgt or Pst. **Figure S4.** Silencing of TaRanGAP2, TaSUN2, or TaWPP1 in N9134R. **Table S1.** SUN proteins identified in wheat. **Table S2.** Potential interacting proteins screened in TaRanGAP2 yeast libraries. **Table S3.** Details of the primers used in this study.

## Data Availability

All data and materials are available in the paper and online supplemental files.

## Abbreviations

CPR5: Constitutive expresser of pathogenesis-related genes 5
DAPs: Differentially accumulated proteins
ETI: Effector-triggered immunity
INM: Inner nuclear membrane
LINC: Linkers of the nucleoskeleton and the cytoskeleton
MAF1: MFP1 attachment factor 1
NE: Nuclear envelope
NETs: NE transmembrane proteins
NLS: Nuclear localization signal
Nups: Nucleoporins
ONM: Outer nuclear membrane
PCD: Programmed cell death
PNS: Perinuclear space
RanGAP: Ran GTPase activating protein
SUN: INM Sad1/UNC-84
TMD: Transmembrane domain
KASH: Klarsicht/ANC-1/Syne-1 homology
SINE: SUN-interacting NE protein
WIP: WPP domain-interacting protein
WIT: WPP domain–interacting tail-anchored protein
