# Supplementary material for: Identification of nuclear membrane SUN proteins and components associated with wheat fungal stress responses
Source: Stress Biol. 2024 Jun 11;4(1):29. doi: 10.1007/s44154-024-00163-z (PMC11166608; doi:10.1007/s44154-024-00163-z)
Supplement: Supplementary file 1 — Additional file 1: Figure S1. Comparison of TaWIT2, TaWIT3 and TaWIT4 with the annotated homologs. Figure S2. Self-activation detection of SUN and KASH proteins. Figure S3. Relative transcript levels of TaRanGAP2, TaSUN2, and TaWPP1 in N9134R/S and 1013R/S inoculated with Bgt or Pst. Figure S4. Silencing of TaRanGAP2, TaSUN2, or TaWPP1 in N9134R. Table S1. SUN proteins identified in wheat. Table S2. Potential interacting proteins screened in TaRanGAP2 yeast libraries. Table S3. Details of the primers used in this study. [file 44154_2024_163_MOESM1_ESM.pdf]

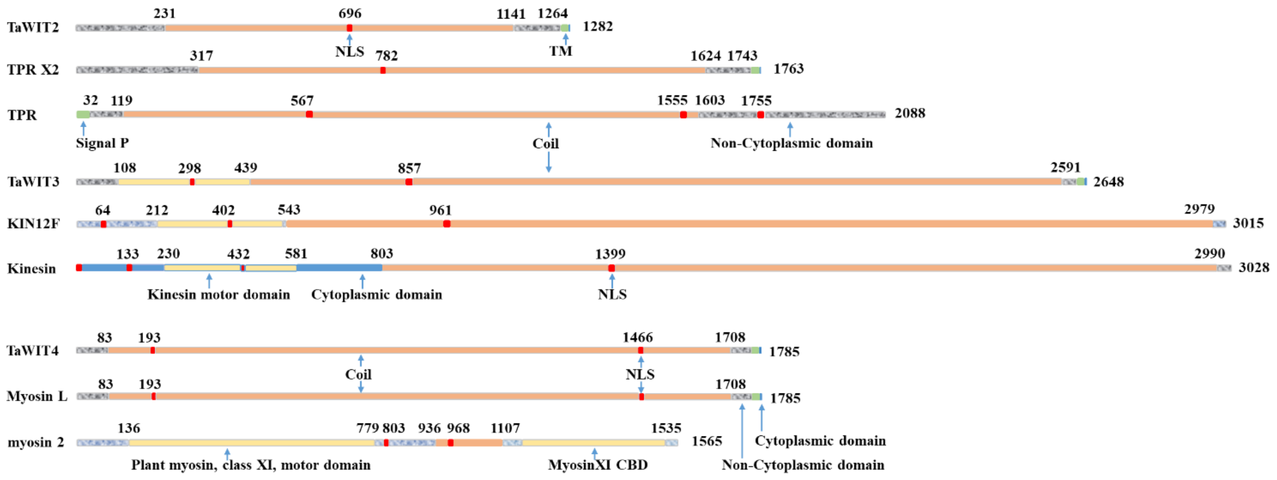

Figure S1. Comparison of TaWIT2, TaWIT3 and TaWIT4 with the annotated homologues, nucleoprotein TPR (EMT01138.1), kinesin (OVA15456.1) and myosin-2 protein (AED95023.1), respectively. The extended coiled-coil domain structures are characterized by orange bar. The transmembrane domains and signal peptide were presented in green. The bipartite nuclear localization signal motif is marked with red. The amino acids that were predicted in cytoplasmic region were shown as blue bar while the non-cytoplasmic region was in grey texture. KIN-12F protein have neither cytosolic region nor non-cytosolic region. The start position of each domain was listed with the corresponding numbers. The length of proteins was given in the right.

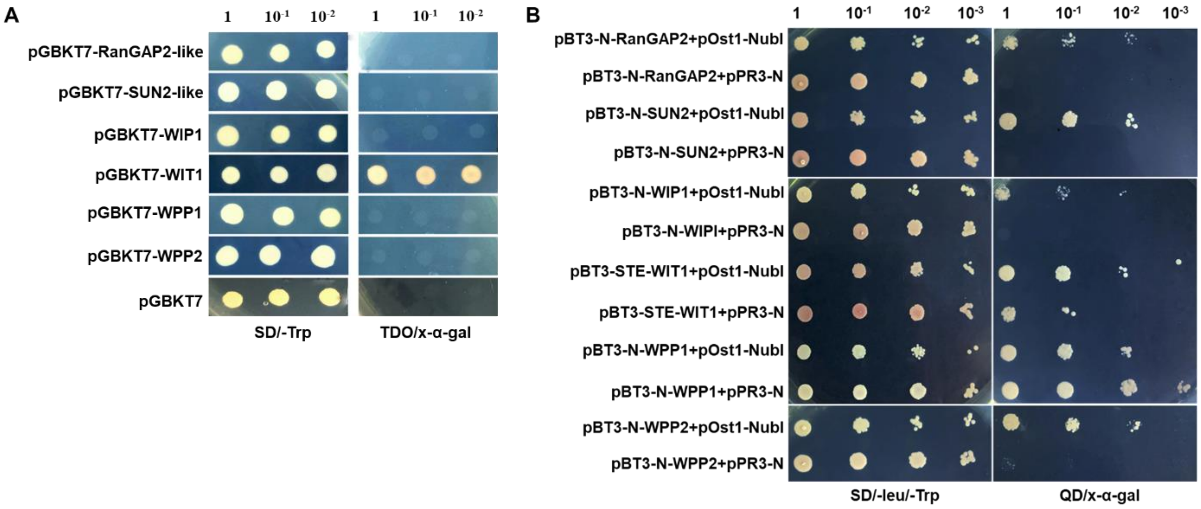

Figure S2. Self-activation detection of SUN and KASH proteins. (A) cloned the full-length CDSs of SUN and KASH into pGBKT7 separately and transferred into yeast. The self-activation of SUN and KASH was detected in TDO/X-α-gal, and pGBKT7 was used as negative control. TDO/X-α-gal, Triple dropout medium without histidine, adenine, and tryptophan. (B) cloned SUN and KASH proteins into pBT3-N or pBT3-STE as baits, pOst1-NubI and pPR3-N as preys were respectively used as positive control and negative control, Prey and bait are transformed in the NMY51 yeast strain and vectors are tested expression and self-activation on QD/X-α-gal.

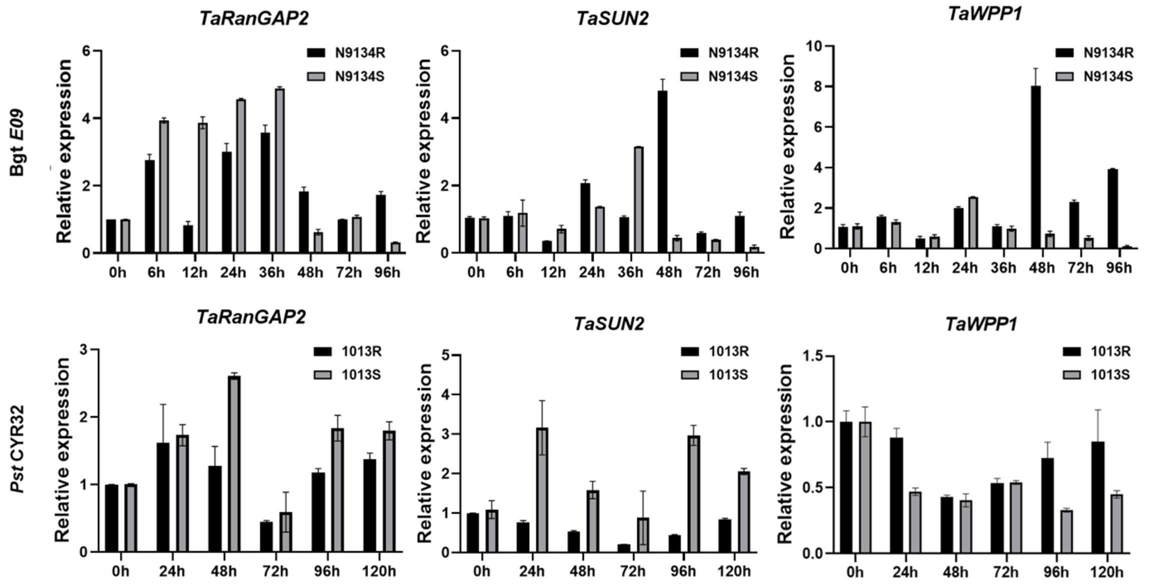

Figure S3. Relative transcript levels of *TaRanGAP2*, *TaSUN2*, and *TaWPP1* in N9134R/S and 1013R/S inoculated with *Bgt* or *Pst*. The y-axis represents the relative gene expression of target gene, while x-axis represents different time points after inoculation. *Actin* was employed as the internal reference. The assays for each gene consisted of at least three biological replicates. Data are means ± standard errors of three independent experiments. Differences between time-course sampling points were assessed using SSPS. *p < 0.05, n = 3.

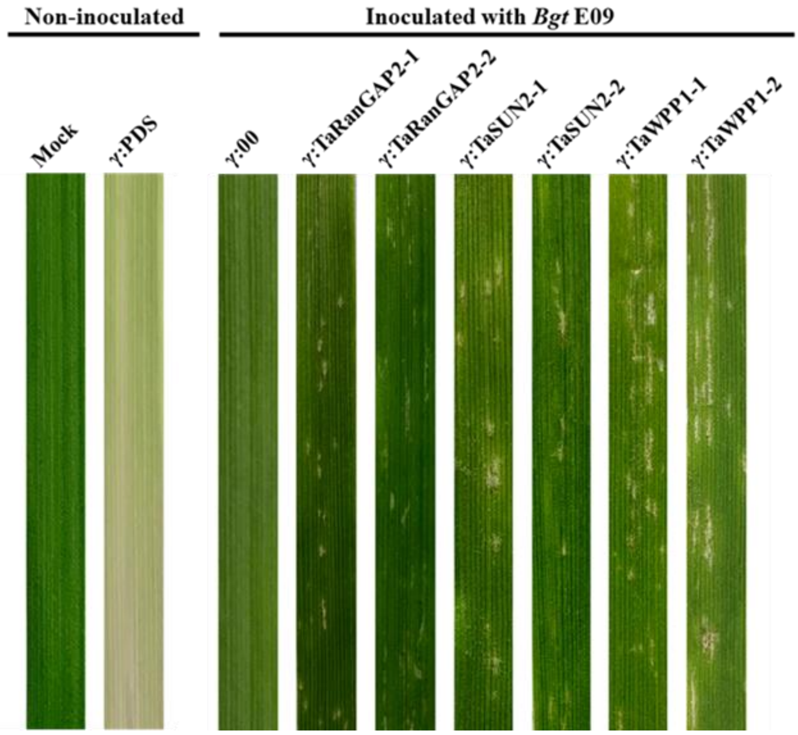

Figure S4. Silencing of *TaRanGAP2*, *TaSUN2*, or *TaWPP1* in leaves of N9134R. Disease phenotypes were taken on the fourth leaves 15 days after inoculated with *Bgt* E09.

Table S1. List of SUN protein identified in wheat

Gene Name	IWGSCV1.1	category	ORF Length(bp)
**TaSUN1**	TraesCS3D02G188400.1	Cter-SUN	1338
	TraesCS3B02G214100.1		1335
	TraesCS3A02G184300.1		1335
**TaSUN2**	TraesCS1D02G090900.1	Cter-SUN	1365
	TraesCS1A02G109200.1		1422
	TraesCS1B02G108700.1		1362
**TaSUN3**	TraesCS3B02G418900.1	Mid-SUN	1959
	TraesCS3D02G379800.1		1956
	TraesCS3A02G389900.1		1836
	TraesCS3A02G389900.2		2019
**TaSUN4**	TraesCS3B02G244900.1	Mid-SUN	1710
	TraesCS3A02G214200.1		1704
	TraesCS3D02G216900.1		1530
	TraesCS3D02G216900.2		1710

Table S2. List of potential interacting proteins screened by TaRanGAP2 in yeast libraries

Screening No.	Gene ID	GenBank	Annotation
**RanGAP+1**	TraesCS4D02G135000	XP_020172226	ribulose bisphosphate carboxylase/oxygenase activase A
**RanGAP+2**	TraesCS4A02G099800	XP_015937582	ATP synthase subunit b'chloroplastic-like
**RanGAP+3**	TraesCS6D02G055400	XP_044418012	60S ribosomal protein L13-1
**RanGAP+4**	TraesCS1D02G105800	XP_044448577	NAD(P)H-quinone oxidoreductase subunit S, chloroplastic-like
**RanGAP+5**	TraesCS1B02G481400	XP_037473898	acetylserotonin O-methyltransferase 1-like
**RanGAP+6**	TraesCS4D02G018500	XP_048569132	TaGRP2 mRNA for glycine-rich RNA-binding protein
**RanGAP+7**	TraesCS2D02G271300	XP_020166780	tyrosine--tRNA ligase, cytoplasmic-like
**RanGAP+8**	TraesCS3B02G433100	XP_037415151	RAN GTPase-activating protein 2-like
**RanGAP+9**	TraesCS7A02G240300	XP_037458347	tricin synthase 1-like
**RanGAP+10**	TraesCS7A02G279500	XP_037459266	zinc finger CCCH domain-containing protein 18-like
**RanGAP+11**	TraesCS2A02G344600	XP_037478056	glyceraldehyde-3-phosphate dehydrogenase A, chloroplastic-like
**RanGAP+12**	TraesCS3A02G413700	XP_037407832	BTB/POZ and TAZ domain-containing protein 2-like
**RanGAP+13**	TraesCS5A02G499300	XP_048532646	expansin-like A1
**RanGAP+14**	TraesCS7D02G016900	XP_048541569	SKP1-like protein 1
**RanGAP+15**	TraesCS2D02G506300	XP_020186519	zinc transporter 8-like
**RanGAP+16**	TraesCS3D02G144900	XP_044355975	protein DJ-1 homolog B-like
**RanGAP+17**	TraesCS2A02G108900	XP_037483558	60S ribosomal protein L19-1-like
**RanGAP+18**	TraesCS3B02G433100	XP_037415151	RAN GTPase-activating protein 2-like
**RanGAP+19**	TraesCS7A02G286800	XP_037459314	AUGMIN subunit 1-like
**RanGAP+20**	TraesCS7A02G222800	XP_037458472	trigger factor-like protein TIG
**RanGAP+21**	TraesCS5D02G425100	AHI44627	ribulose-1,5-bisphosphate carboxylase/oxygenase large subunit
**RanGAP+22**	TraesCS6A02G358100	XP_037448725	photosystem II 10 kDa polypeptide, chloroplastic-like
**RanGAP+23**	TraesCS5D02G425100	XP_048562098	ribulose bisphosphate carboxylase large chain-like

Screening No.	Gene ID	GenBank	Annotation
**RanGAP+24**	TraesCS6D02G378000	XP_044421699	CBS domain-containing protein CBSX2, chloroplastic-like
**RanGAP+25**	TraesCS4B02G332800	XP_044371599	ABA-inducible protein PHV A1-like
**RanGAP+26**	TraesCS2B02G234500	XP_037484585	cytochrome b6-f complex iron-sulfur subunit, chloroplastic-like
**RanGAP+27**	TraesCS3B02G433100	XP_044351265	RAN GTPase-activating protein 2-like
**RanGAP+28**	TraesCS7B02G019600	XP_044435324	thiosulfate sulfurtransferase 16, chloroplastic-like
**RanGAP+29**	TraesCS6A02G418300	XP_048537103	low-temperature-induced 65 kDa protein-like
**RanGAP+30**	TraesCS1B02G432700	XP_037427093	chlorophyll a-b binding protein of LHCII type 1-like
**RanGAP+31**	TraesCS5B02G240600	XP_044389114	tuliposide A-converting enzyme 2, chloroplastic-like
**RanGAP+32**	TraesCS4D02G344300	XP_020150374	probable aquaporin TIP4-1
**RanGAP+33**	TraesCS2B02G078900	XP_037475487	ribulose bisphosphate carboxylase small chain PWS4.3, chloroplastic-like
**RanGAP+34**	TraesCS7D02G133500	XP_020174026	probable galactinol--sucrose galactosyltransferase 6
**RanGAP+35**	none	KAF2188070	transmembrane amino acid transporter protein-domain-containing protein
**RanGAP+36**	TraesCS1D02G428800	XP_044984444	solanesyl-diphosphate synthase 2, chloroplastic-like
**RanGAP+37**	TraesCS5D02G099500	XP_020193135	probable prefoldin subunit 3
**RanGAP+38**	TraesCS4D02G118500	XP_020184643	CBL-interacting protein kinase 15-like
**RanGAP+39**	TraesCS3B02G433100	XP_037415151	RAN GTPase-activating protein 2-like
**RanGAP+40**	TraesCS2A02G206200	XP_037484574	chlorophyll a-b binding protein CP29.2, chloroplastic-like
**RanGAP+41**	TraesCS3B02G377300	XP_044350688	Werner Syndrome-like exonuclease
**RanGAP+42**	TraesCS4A02G290600	XP_037422485	E3 ubiquitin-protein ligase RNF5-like
**RanGAP+43**	TraesCS1A02G386300	EMS52199	60S ribosomal protein L37a-1
**RanGAP+44**	TraesCS3D02G239800	XP_044354552	Werner Syndrome-like exonuclease
**RanGAP+45**	TraesCS1A02G420900	XP_044348608	solanesyl-diphosphate synthase 2, chloroplastic-like
**RanGAP+46**	TraesCS1A02G080600	XP_044459016	CBL-interacting protein kinase 17-like
**RanGAP+47**	TraesCS6D02G230600	XP_044420261	peroxisome biogenesis protein 19-1-like
**RanGAP+48**	TraesCS7D02G239500	XP_044957449	vulgare proteoglycan 4-like

Table S3. The detailed information regarding primers used for the article

Primer	Forward primer sequence	Reverse primer sequence
**TaRanGAP2**	GAAGTGCCACGTGTCCACATGG	TGCGAAATTCCAGCCGGT
**TaSUN2**	GATCGGTTACCCCCTTCGTC	AGTCAGCGTCTGTCCATAGTG
**TaWIP1**	TGCCAGAACGACCATAACGAT	ATAAGGCCACCAATGCCTAATA
**TaWIT1**	ATGCGACTTTCATGTGTAGG	TGAACACTAGCACAGAACTC
**TaWPP1**	CTCTCCTCCCACTCCACGC	AGAGGCTGTCACTGATCTTAC
**TaWPP2**	CCGTGGCACGCGGCCGCGAG	CTGAGAGGCACAATGTTCTG
**TaSUN1**	CGATTCGATCCCCACCCTA	GCCCTAAAGCAAAACAGATACAAA
**pYJ:GFP-TaRanGAP2**	TAGCCATGGTAGATCTGATGGCTTCAGCGGCGCAAG	GCCTTACGTAACTAGTGTCCTCCTCCGCCTC
**pYJ:GFP-TaSUN2**	TAGCCATGGTAGATCTGATGGCTTCAACTGCTGC	GCCTTACGTAACTAGTCGATGCCGCAGTGCCG
**pYJ:GFP-TaSUN1**	TAGCCATGGTAGATCTGATGCCTGCAGGTCGACG	GCCTTACGTAACTAGTGGGCAGTGGACTAG
**pYJ:GFP-TaWIP1**	TAGCCATGGTAGATCTGATGGACTCCGGCGCCAAC	GCCTTACGTAACTAGTGGCCATGCGCCCTTC
**pYJ:GFP-TaWIT1**	TAGCCATGGTAGATCTGATGAGTGCCGAGAACAC	GCCTTACGTAACTAGTCAAGATGCTGCCTAC
**pYJ:GFP-TaWPP1**	TAGCCATGGTAGATCTGATGGCCGAGGACGCCC	GCCTTACGTAACTAGTCTCCTCCGTGGGGGGCGGAG
**pYJ:GFP-TaWPP2**	TAGCCATGGTAGATCTGATGGGCCCCGACGAGCTC	GCCTTACGTAACTAGTGGCCTGGGAGGTCTC
**pGBKT7-TaRanGAP2**	CATGGAGGCCGAATTCATGGCTTCAGCGGCGCAAG	CTGCAGGTCGACGGATCTAGTCCTCCTCCGCCTC
**pGBKT7-TaSUN2**	CATGGAGGCCGAATTCATGGCTTCAACTGCTGC	CTGCAGGTCGACGGATTCACGATGCCGCGGTGCCG
**pGBKT7-TaWIP1**	CATGGAGGCCGAATTCATGGACTCCGGCGCCAAC	CTGCAGGTCGACGGATTCAAGTAGGTACAAATTC
**pGBKT7-TaWIT1**	CATGGAGGCCGAATTCATGAGTGCCGAGAACAC	CTGCAGGTCGACGGATTCAGGCCATGCGCCCTTC
**pGBKT7-TaWPP1**	CATGGAGGCCGAATTCATGGCCGAGGACGCCC	CTGCAGGTCGACGGATTTACTCCTCCGTGGG
**pGBKT7-TaWPP2**	CATGGAGGCCGAATTCATGGGCCCCGACGAGCTC	CTGCAGGTCGACGGATTCAGGCCTGGGAGGTCTC
**pGBKT7-TaSUN1**	CATGGAGGCCGAATTCATGCCTGCAGGTCGACG	CTGCAGGTCGACGGATTCAGGGCAGTGGACTAG
**pGADT7-TaRanGAP2**	GGAGGCCAGTGAATTCATGGCTTCAGCGGCGCAAG	CTCGAGCTCGATGGATCTAGTCCTCCTCCGCCTC
**pGADT7-TaSUN2**	GGAGGCCAGTGAATTCATGGCTTCAACTGCTGC	CTCGAGCTCGATGGATTCACGATGCCGCGGTGCCG

**pGADT7-TaWIP1**	GGAGGCCAGTGAATTCATGGACTCCGGCGCCAAC	CTCGAGCTCGATGGATTCAAGTAGGTACAAATTC
**pGADT7-TaWIT1**	GGAGGCCAGTGAATTCATGAGTGCCGAGAACAC	CTCGAGCTCGATGGATTCAGGCCATGCGCCCTTC
**pGADT7-TaWPP1**	GGAGGCCAGTGAATTCATGGCCGAGGACGCCC	CTCGAGCTCGATGGATTTACTCCTCCGTGGG
**pGADT7-TaWPP2**	GGAGGCCAGTGAATTCATGGGCCCCGACGAGCTC	CTCGAGCTCGATGGATTCAGGCCTGGGAGGTCTC
**pGADT7-TaSUN1**	GGAGGCCAGTGAATTCATGCCTGCAGGTCGACG	CTCGAGCTCGATGGATTCAGGGCAGTGGACTAG
**pPR3-N-TaRanGAP2**	AGAGTGGCCATTACGGCCATGGCTTCAGCGGCGCAAG	GAGAGGCCGAGGCGGCCGCTAGTCCTCCTCCGCCTC
**pPR3-N-TaSUN2**	AGAGTGGCCATTACGGCCATGGCTTCAACTGCTGC	GAGAGGCCGAGGCGGCCGTCACGATGCCGCAGTGCCG
**pPR3-N-TaWIP1**	AGAGTGGCCATTACGGCCATGGACTCCGGCGCCAAC	GAGAGGCCGAGGCGGCCGTCAAGTAGGTACAAATTC
**pPR3-N-TaWIT1**	AGAGTGGCCATTACGGCCATGAGTGCCGAGAACAC	GAGAGGCCGAGGCGGCCGTCAGGCCATGCGCCCTTC
**pPR3-N-TaWPP1**	AGAGTGGCCATTACGGCCATGGCCGAGGACGCCC	GAGAGGCCGAGGCGGCCGTTACTCCTCCGTGGG
**pPR3-N-TaWPP2**	AGAGTGGCCATTACGGCCATGGGCCCCGACGAGCTC	GAGAGGCCGAGGCGGCCGTCAGGCCTGGGAGGTCTC
**pBT3-N-TaRanGAP2**	TGCAGGGCCATTACGGCCATGGCTTCAGCGGCGCAAG	CCATGGGGCCGAGGCGGCCTAGTCCTCCTCCGCCTC
**pBT3-N-TaSUN2**	TGCAGGGCCATTACGGCCATGGCTTCAACTGCTGC	CCATGGGGCCGAGGCGGCTCACGATGCCGCAGTGCCG
**pBT3-N-TaWIP1**	TGCAGGGCCATTACGGCCATGGACTCCGGCGCCAAC	CCATGGGGCCGAGGCGGCTCAAGTAGGTACAAATTC
**pBT3-STE-TaWIT1**	GTAATGGCCATTACGGCCATGAGTGCCGAGAACAC	GCAGATGGCCGAGGCGGCTCAGGCCATGCGCCCTTC
**pBT3-N-TaWPP1**	TGCAGGGCCATTACGGCCATGGCCGAGGACGCCC	CCATGGGGCCGAGGCGGCTTACTCCTCCGTGGG
**pBT3-N-TaWPP2**	TGCAGGGCCATTACGGCCATGGGCCCCGACGAGCTC	CCATGGGGCCGAGGCGGCTCAGGCCTGGGAGGTCTC
**cLUC-SUN2**	GTCTTACCGGAAAACTCGATGGCTTCAACTGCTGC	AAAGCTCTGCAGGTCGACTCACGATGCCGCGGTGCCG
**cLUC-WPP1**	GTCTTACCGGAAAACTCGATGGCCGAGGACGCCC	AAAGCTCTGCAGGTCGACTTACTCCTCCGTGGG
**WIP1-nLUC**	GGGGACGAGCTCGGTACCATGGACTCCGGCGCCAAC	GTACGAGATCTGGTCGACAGTAGGTACAAATTC
**WIT1-nLUC**	GGGGACGAGCTCGGTACCATGAGTGCCGAGAACAC	GTACGAGATCTGGTCGACGGCCATGCGCCCTTC
**cEYFP-TaSUN1**	CGAGCTCAAGCTTCGAAATGCCTGCAGGTCGACG	CGACTGCAGAATTCGAATCAGGGCAGTGGACTAG
**cEYFP-TaSUN2**	CGAGCTCAAGCTTCGAAATGGCTTCAACTGCTGC	CGACTGCAGAATTCGAATCACGATGCCGCAGTGCCG
**cEYFP-TaRanGAP2**	CGAGCTCAAGCTTCGAAATGGCTTCAGCGGCGCAAG	CGACTGCAGAATTCGAACTAGTCCTCCTCCGCCTC
**cEYFP-TaWIP1**	CGAGCTCAAGCTTCGAAATGGACTCCGGCGCCAAC	CGACTGCAGAATTCGAATCAAGTAGGTACAAATTC

**cEYFP-TaWIT1**	CGAGCTCAAGCTTCGAAATGAGTGCCGAGAACAC	CGACTGCAGAATTCGAATCAGGCCATGCGCCCTTC
**cEYFP-TaWPP1**	CGAGCTCAAGCTTCGAAATGGCCGAGGACGCCC	CGACTGCAGAATTCGAATTACTCCTCCGTGGG
**cEYFP-TaWPP2**	CGAGCTCAAGCTTCGAAATGGGCCCCGACGAGCTC	CGACTGCAGAATTCGAATCAGGCCTGGGAGGTCTC
**nEYFP-TaSUN1**	CGAGCTCAAGCTTCGAAATGCCTGCAGGTCGACG	CGACTGCAGAATTCGAATCAGGGCAGTGGACTAG
**nEYFP-TaSUN2**	CGAGCTCAAGCTTCGAAATGGCTTCAACTGCTGC	CGACTGCAGAATTCGAATCACGATGCCGCAGTGCCG
**nEYFP-TaRanGAP2**	CGAGCTCAAGCTTCGAAATGGCTTCAGCGGCGCAAG	CGACTGCAGAATTCGAACTAGTCCTCCTCCGCCTC
**nEYFP-TaWIP1**	CGAGCTCAAGCTTCGAAATGGACTCCGGCGCCAAC	CGACTGCAGAATTCGAATCAAGTAGGTACAAATTC
**nEYFP-TaWIT1**	CGAGCTCAAGCTTCGAAATGAGTGCCGAGAACAC	CGACTGCAGAATTCGAATCAGGCCATGCGCCCTTC
**nEYFP-TaWPP1**	CGAGCTCAAGCTTCGAAATGGCCGAGGACGCCC	CGACTGCAGAATTCGAATTACTCCTCCGTGGG
**nEYFP-TaWPP2**	CGAGCTCAAGCTTCGAAATGGGCCCCGACGAGCTC	CGACTGCAGAATTCGAATCAGGCCTGGGAGGTCTC
**BSMV-TaRanGAP2**	TTCTTCCGTTTCTAAGTAACTGCGGAACCAGAA	CTTGCTATTTCTAGCTCTAAGTATCTCAGTACAGAC
**BSMV-TaSUN2**	TTCTTCCGTTTCTAAGTAAGAGCATTCTTCCTCGTC	CTTGCTATTTCTAGCTCTAAAGGTCTGGGCGTTG
**BSMV-TaWIP1**	TTCTTCCGTTTCTAAGTAATGACAATTCCACAGACAA	CTTGCTATTTCTAGCTCTAAAGTGCCCTGACCCTC
**BSMV-TaWIT1**	TTCTTCCGTTTCTAAGTAACATACAGGTCCATTTAA	CTTGCTATTTCTAGCTCTAACTTCTCCAGCAACAA
**BSMV-TaWPP1**	TTCTTCCGTTTCTAAGTAAGTCGCTCAGCATCTGGC	CTTGCTATTTCTAGCTCTAAACGAACCCTCCGACACTT
**BSMV-TaWPP2**	TTCTTCCGTTTCTAAGTAAGCCCCGTTCTCCTTCAG	CTTGCTATTTCTAGCTCTAAGCGGAGCGGGACTTGA
**q-TaRanGAP2**	GCAGCCTGTTTTGGTGTAGC	TCGACCAGAGAACCAGGACA
**q-TaSUN2**	CAGCGGATGGTACCAAGGAG	CGTAAGTGAACTCCCCCAGG
**q-TaWIP1**	TTCACGAAGCGCAGAAGACT	TGAAGCGTTCGACAGCTCTT
**q-TaWIT1**	GAGAACGGCTTCATCTCCAGA	GGCCAGTGTAGCGAACTGAA
**q-TaWPP1**	CCCTTGCTCTCCACACCATG	TGCTGAGCGACGGAAACAG
**q-TaWPP2**	TTGTTCTTTCAGTAGCTGCT	TCCGGTCAAATCTTGGATCA
**q-TaBRI1**	GGTGGACTCGAAAACCTCGT	CTCCTTGGCTTCCTTGAGGG
**q-TaBAK2**	TGATGGTGGCTGCTGCTAAA	TGAACAAATGGGGGATCGCA
**q-TaSTK**	AAGGATCTAATGTCGGTCGTCAAG	ACTCGTGCTGCCAATCCAAA

**q-Ta14-3-3**	CACCTTGTCCCGTCTTCCA	TCTCAGCAGCATCCTTCCTCT
**q-TaBZR1**	CGGCACCCGTTCTTCGCCCTAT	CTGATCCACCGGCCAGAGTCCA
**q-TaPR1**	ATCTGTGAAAGCAACGCGTG	CAACTTCGCAGGTTACGCTC
